# The relationship between high-density lipoprotein cholesterol (HDL-C) and glycosylated hemoglobin in diabetic patients aged 20 or above: a cross-sectional study

**DOI:** 10.1186/s12902-021-00863-x

**Published:** 2021-10-11

**Authors:** Rui Huang, Li Yan, Yuhua Lei

**Affiliations:** 1 Cardiovascular Disease Center, Central Hospital of Tujia and Miao Autonomous Prefecture, Enshi Clinical College of Wuhan University, No.158 Wuyang Avenue, Enshi City, 445000 Hubei Province China; 2 Pediatrics Department, Central Hospital of Tujia and Miao Autonomous Prefecture, Enshi Clinical College of Wuhan University, No.158 Wuyang Avenue, Enshi City, 445000 Hubei Province China

**Keywords:** High-density lipoprotein cholesterol, HDL-C, Glycosylated hemoglobin, Diabetes

## Abstract

**Aim:**

The incidence rate of diabetes is increasing year by year, seriously threatening human health. As a predictor of glycemic control, glycated hemoglobin is reported to be related to various complications and prognoses of diabetes. Besides, HDL-C dyslipidemia is a component of metabolic syndrome and may be related to various cardiovascular and cerebrovascular diseases. The principal objective of this project was to investigate the relationship between HDL-C and glycosylated hemoglobin in adult diabetic patients.

**Methods:**

A total of 3171 adult diabetic patients aged 20 years and above were included in the present study from the National Health and Nutrition Examination Survey (NHANES). HDL-C and glycosylated hemoglobin were regarded as independent and dependent variables, respectively. EmpowerStats software and R (version 3.4.3) were used to examine the association between HDL-C and glycosylated hemoglobin.

**Results:**

HDL-C was inversely associated with glycohemoglobin after adjusting for other covariates (β = − 0.004, 95% CI:− 0.008 to − 0.000, *p* = 0.044). Race/ethnicity and age were considered the most prominent interactive factors that affect the relationship between HDL and glycosylated hemoglobin by the interaction analysis. A U-shaped association was detected between HDL-C and glycosylated hemoglobin for people of other race/ethnicity or aged 60 and above, which had an inflection point of HDL-C at 60 mg/dL. In contrast, we observed an inverted U-shaped distribution between HDL-C and glycosylated hemoglobin in people under 40 with point of inflection located at 60 mg/dL as well.

**Conclusions:**

HDL-C in diabetic patients is inversely associated with glycosylated hemoglobin and may be relevant to glycemic control. However, a U-shaped relationship was also observed in a certain kind of people, which implied that, though HDL-C is considered as metabolism and anti-atherogenic property, for diabetics, it is not the higher, the better.

**Supplementary Information:**

The online version contains supplementary material available at 10.1186/s12902-021-00863-x.

## Introduction

Diabetes Mellitus (DM) is a complex chronic disease characterized by high prevalence, morbidity, and excess mortality, especially in the middle-aged and elderly. Currently, 425 million adult population worldwide are diagnosed with DM, which has posed unprecedented challenges and economic burdens to the global medical and healthy industries [1, 2].

Although various approaches have been used in managing DM, the primary goal is to achieve and maintain optimal glycemic control and delay the onset of diabetes-related complications such as cardiovascular diseases, renal insufficiency, peripheral neuropathy, macrovascular and microvascular disorders. Extensive research has shown that glycosylated hemoglobin or HbA1C is recognized as a pivotal sign to evaluate the long-term serum glucose control and complications of diabetes currently, which is unlikely to be supplanted in the short term [3–7]. However, HbA1C depicts a 2-3-month average plasma glucose concentration and may not capture daily blood glucose swings or variability during patients’ daily lives [1, 8, 9]. According to a recent report, the variability of HbA1c in diabetic patients is related to cardiovascular events and all-cause mortality, and high HbA1c is independently associated with an increased risk of microvascular complications [10]. Consistent with this research, other studies also found that high HbA1c was associated with increased cardiovascular death [11] and metabolic syndrom [12, 13].

HDL-C is a highly effective biomarker, which is considered to be inversely correlated with the risk of cardiovascular diseases and plays a crucial role in predicting adverse results [14–17]. However, as reported in the relevant literature, the classic HDL-C theory defined as the notion that intervention to increase HDL-C concentrations will vastly lessen the occurrence of adverse events is now being questioned [1, 15, 16, 18, 19]. More importantly, it is still unclear what HDL-C concentration in DM can minimize cardiovascular risk occurrence [15]. Therefore, it is of great significance to analyze and realize the relationship between HDL-C and glycosylated hemoglobin. Besides, to the best of our knowledge, there is no study on the association between glycosylated hemoglobin and HDL-C in adults aged 20 years and over with DM in the NHANES population.

The ultimate purpose of this paper is to analyze the relationship between glycohemoglobin and HDL-C, to provide crucial clinical significance for the management of serum cholesterol, especially HDL-C, in diabetes mellitus patients, and to find a possible alternative to HbA1c to reflect the glycaemic control effectively.

## Methods

### Study population

Our research used the NHANES data collected from 1999 to 2018, which contains cross-sectional socio-demographic, dietary, and medical data collected through questionnaires, standardized physical examinations, and laboratory tests. NHANES is a population-based research program conducted by the National Center for Health Statistics (NCHS) to monitor the health and nutritional status of civilians and non-medical personnel in the United States. A multi-stage, complex clusters, probabilistic design is adopted for data collection and analysis, rather than a simple random sample based on the US population [20–22].

A total of 6807 cases who reported having DM were included in the primary analysis. The inclusion criteria were subjects aged 20 or above who were diagnosed with DM. We excluded certain participants as follows:(1) individuals with missing glycohemoglobin or HDL-C or laboratory tests;(2) those with regular alcohol consumption, cancer, liver disfunction, cronic kidney disease, hereditary hyperlipidemia;(3) those who were overweight or obese with BMI over 24 kg/m^2^. NCHS Ethics Review Board supported the research. Furthermore, written informed consent was received from each subject [23].

### Variables

The main variables of this study were glycohemoglobin (dependent variable) and HDL-C (independent variable). Both HDL-C and glycohemoglobin were tested and recorded in authoritative laboratories using standard procedures. See the supplementary information for specific testing equipment and laboratories.

Besides, the following variables were included in the present study: age, race/ethnicity, sex, body mass index (BMI), waist circumference, systolic blood pressure (SBP), diastolic blood pressure (DBP), alanine aminotransferase (ALT), creatinine (Cr), triglyceride (TG), total cholesterol (TC), LDL-cholesterol (LDL), plasma fasting glucose (FDG), gamma-glutamyl transferase (γGT), serum uric acid (sUA) and insulin. In addition, prescription drugs including lipid-lowering medications and anti-diabetic medications were also collected and analyzed.

For blood pressure measurement: After 5 min of resting quietly in the seat, once the participant’s maximum inflation level (MIL) is determined, three consecutive blood pressure readings will be obtained. If the blood pressure measurement is interrupted or incomplete, a fourth attempt can be made. All blood pressure measurements (systolic and diastolic blood pressure) are performed in the Mobile Inspection Center (MEC). The absolute blood pressure is the average of three valid measurements.

We excluded subjects with missing independent or dependent variables. For missing continuous variables, we use the median to fill in. For missing categorical variables, we separate the missing group as a group. All the covariate acquisition processes and any detailed information can be found at www.cdc.gov/nchs/nhanes/.

### Statistical analyses

The statistical analyses were conducted using package R (version 3.4.3, http://www.Rproject.org) and EmpowerStats software (http://www.empowerstats.com). A two-sided *p* < 0.05 was considered to be statistically significant. We used the weighted analysis as recommended by the NCHS Analysis Guide to maintain national representation. The continuous variables were characterized by mean ± standard deviation, or as median and interquartile range, as appropriate. The categorical variables were presented as a percentage. The *P*-value was calculated using a weighted chi-squared test for categorical variables and a weighted linear regression model for continuous variables. The association between HDL-C and HbA1c was examined using multivariable linear regression. To further analyze the relationship between HDL-C and HbA1c, we used the following three models: Model 1: No adjustment for variables; Model 2: Adjusted for sex, age, and race/ethnicity; Model 3: Adjusted for sex, age, race/ethnicity, BMI, waist circumference, systolic blood pressure, diastolic blood pressure, ALT, Cr, TG, TC, LDL, FDG, γGT, uric acid, insulin, and lipid-lowering medications and anti-diabetic medications.

An interaction test was conducted to evaluate whether patients’ characteristics influence the association between HDL and Hba1c. In addition to further research on data, we performed subgroup analysis subsequently. A weighted generalized additive model and a smooth curve fitting were performed to address nonlinearity between HDL-C and glycohemoglobin. When nonlinearity was discovered, we first calculated the vital inflection point using a recursive algorithm. We then performed a weighted two-piecewise linear regression model on both sides of the inflection point.

## Results

Table 1 shows the general description of weighed characteristics of all 3171 subjects included in the study. Of all these participants, the average age was 46.86 ± 16.80 years old,47.88% were male, 52.12% were female, 8.17% were Mexican Americans, 68.03% were Non-Hispanic Whites, 10.70% were Non-Hispanic Blacks, and 13.10% were other race/ethnicity. Among all subjects, there were 232 cases with glycosylated hemoglobin greater than or equal to 7.0%, and 343 cases with greater than or equal to 6.5%. Among the two groups stratified by glycohemoglobin (<7,> = 7), age, ALT, γGT, Glu, TG,HDL-C, BMI, waist circumference, SBP, DBP, age groups (age < 40 years,> = 40, < 60 years and > =60 years), lipid-lowering medications and anti-diabetic medications were all of great statistical significance(*p* < 0.05).

**Table 1:** Description of the participants included in the study

Glycohemoglobin	< 7 *N* = 2939	≥7 *N* = 232	All *N* = 3171	*P*-value
Age (years)	46.27 ± 16.77	57.37 ± 13.53	46.86 ± 16.80	< 0.0001
ALT(U/L)	25.85 ± 46.66	33.63 ± 25.83	26.26 ± 45.83	0.0329
Cr (umol/L)	77.13 ± 29.41	77.96 ± 26.38	77.18 ± 29.26	0.7215
γGT (IU/L)	28.49 ± 40.77	44.17 ± 43.89	29.32 ± 41.09	< 0.0001
Glu (mg/dL)	93.54 ± 16.56	191.86 ± 70.91	98.73 ± 31.76	< 0.0001
sUA (mg/dL)	5.35 ± 1.46	5.39 ± 1.25	5.35 ± 1.45	0.7932
TG (mg/dL)	120.71 ± 85.30	187.58 ± 203.12	124.78 ± 97.97	< 0.0001
LDL (mg/dL)	116.11 ± 34.71	109.60 ± 37.31	115.74 ± 34.90	0.0945
TC (mg/dL)	195.14 ± 40.41	199.99 ± 77.05	195.39 ± 43.14	0.1563
HDL-C (mg/dL)	53.79 ± 16.25	44.84 ± 12.93	53.31 ± 16.22	< 0.0001
glycohemoglobin (%)	5.42 ± 0.43	8.53 ± 1.54	5.58 ± 0.89	< 0.0001
BMI (kg/m^2^)	28.20 ± 6.32	32.12 ± 7.28	28.42 ± 6.43	< 0.0001
Waist circumference (cm)	96.38 ± 14.98	108.57 ± 16.76	97.02 ± 15.32	< 0.0001
SBP (mmHg)	118.39 ± 16.41	118.62 ± 14.41	118.40 ± 16.31	0.8585
DBP (mmHg)	66.08 ± 13.28	64.75 ± 15.08	66.01 ± 13.39	0.2121
Sex(%)				0.0906
Male	47.53	54.24	47.88	
Female	52.47	45.76	52.12	
Race/ethnicity (%)				0.0598
Mexican American	7.88	13.30	8.17	
Other race/ethnicity	13.09	13.32	13.10	
Non-Hispanic White	68.42	61.05	68.03	
Non-Hispanic Black	10.61	12.33	10.70	
Age groups (%)				< 0.0001
Age < 40 years	39.82	8.61	38.17	
Age > =40, < 60 years	36.37	42.58	36.70	
Age > =60 years	23.81	48.81	25.13	
Medications				
Lipid-lowering medications	74.17	33.23		< 0.0001
Anti-diabetic medications	73.43	36.68		< 0.0001

The continuous variables were characterized by mean ± standard and analyzed using a weighted linear regression model for continuous variables. The categorical variables were presented as percentages, and the *p-*value was calculated using a weighted chi-squared test.

**Fig. 1:**
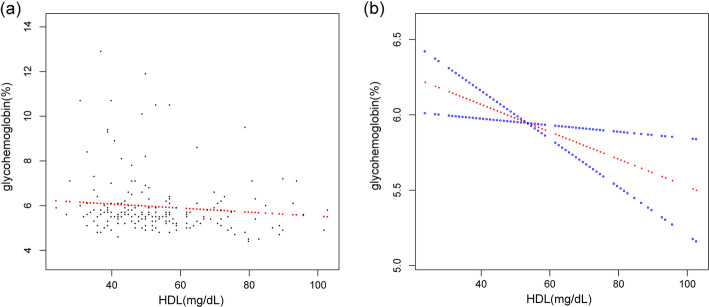
Scatter plot of the distribution of HDL-C and glycohemoglobin. Each black point represents a sample(a). The red line represents the smooth curve fit between variables. In comparison, blue bands represent the 95% CI(b). Sex, age, race/ethnicity, BMI, waist circumference, systolic blood pressure, diastolic blood pressure, ALT, Cr, TG, TC, LDL, FDG, γGT, uric acid,insulin, lipid-lowering medications and anti-diabetic medications were adjusted

We observed a negative relationship between HDL-C and glycohemoglobin in the fully-adjusted model (β = − 0.004, 95% CI:-0.008 to − 0.000, *p* = 0.044). The trend remained to be of statistical significance among the HDL-C tertile groups(*p* < 0.001). In sub-analysis stratified by age, race/ethnicity, this negative association was observed only in males [β = − 0.015,95% CI: − 0.026 to − 0.004, *p* = 0.008],and in non-Hispanic White [β = − 0.008, 95% CI:-0.014, − 0.001, *p* = 0.025] and in the elderly who were aged 60 years or above (β = − 0.016, 95% CI:-0.029 to − 0.002,*p* = 0.027). Race/ethnicity and age were considered the most prominent interactive factors that affect the relationship between HDL and glycosylated hemoglobin by the interaction analysis. (Table 2, Fig. 1).

**Table 2:** Association between glycohemoglobin (%) and HDL-C (mg/dL)

	Model 1, β(95% CI) , *p*	Model 2, β(95% CI) , *p*	Model 3, β(95% CI) , *p*	P for interaction
HDL-C	-0.008, (− 0.010, − 0.007), < 0.001	− 0.010,(− 0.012, − 0.008),< 0.001	− 0.004,(− 0.008, − 0.000),0.044	
**Tertiles of HDL-C**				
Lowest tertile	Reference	Reference	Reference	
Q2	−0.161, (− 0.237, − 0.085),< 0.001	−0.164,(− 0.236, − 0.091),< 0.001	−0.016,(− 0.042, − 0.003),0.006	
Q3	−0.328, (− 0.403, − 0.252),< 0.001	− 0.382, (− 0.458, − 0.307), < 0.001	− 0.070,(− 0.210, − 0.017),0.033	
*P* for trend	< 0.001	< 0.001	< 0.001	
**Stratified by sex**				**0.923**
Male	−0.010, (− 0.013, − 0.007),< 0.001	− 0.011,(− 0.014, − 0.008),< 0.001	− 0.009,(− 0.016, − 0.007), < 0.001	
Female	− 0.009,(− 0.011, − 0.006),< 0.001	− 0.010,(− 0.012, − 0.007),< 0.001	− 0.002,(− 0.007, 0.003), 0.551	
**Stratified by race/ethnicity**				0.027
Mexican American	−0.008, (− 0.015, − 0.001), 0.022	−0.013,(− 0.020, − 0.006),< 0.001	0.008,(− 0.005, 0.022),0.248	
Other Race/ethnicity	−0.011, (− 0.016, − 0.006), < 0.001	− 0.010,(− 0.015, − 0.004),< 0.001	− 0.005,(− 0.014, 0.004),0.307	
Non-Hispanic White	− 0.008, (− 0.011, − 0.006), < 0.001	−0.011, (− 0.013, − 0.008), < 0.001	−0.008,(− 0.014, − 0.001),0.025	
Non-Hispanic Black	−0.007,(− 0.012, − 0.002),0.004	−0.008,(− 0.013, − 0.004),< 0.001	−0.008,(− 0.017, 0.007), 0.054	
**Stratified by age**				0.023
Age < 40 years	−0.005,(− 0.008, − 0.003), < 0.001	−0.005, (− 0.008, − 0.003),< 0.001	−0.003(− 0.009,0.004)0.447	
Age > =40, < 60 years	−0.010,(− 0.014, − 0.007),< 0.001	−0.011,(− 0.014, − 0.007),< 0.001	−0.004(− 0.010,0.003)0.258	
Age > =60 years	− 0.014,(− 0.017, − 0.011),< 0.001	− 0.015, (− 0.018, − 0.012) < 0.001	− 0.008(− 0.017,0.001)0.092	

Model 1: No adjustment for variables;

Model 2: Sex, age, and race/ethnicity were adjusted;

Model 3: Sex, age, race/ethnicity, BMI, waist circumference, systolic blood pressure, diastolic blood pressure, ALT, Cr, TG, TC, LDL, FDG, γGT, uric acid, insulin and lipid-lowering medications and anti-diabetic medications were adjusted.

**Fig. 2:**
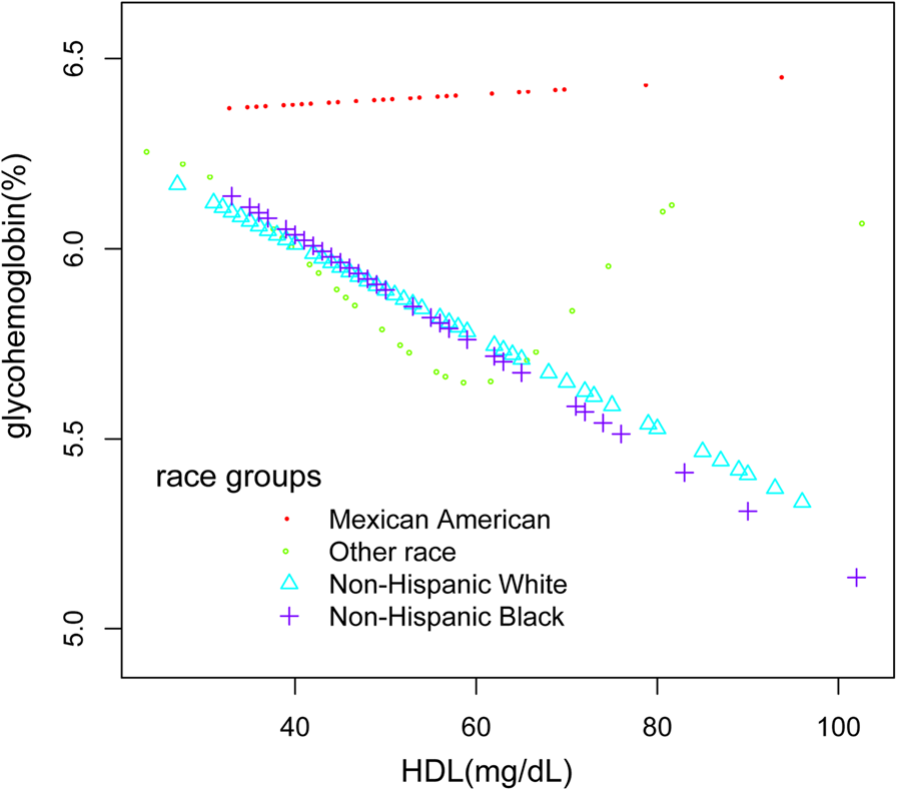
The association between HDL-C and glycohemoglobin stratified by race. Each line represents the smooth curve fit between variables. Sex, age, race/ethnicity, BMI, waist circumference, systolic blood pressure, diastolic blood pressure, ALT, Cr, TG, TC, LDL, FDG, γGT, uric acid, insulin, lipid-lowering medications and anti-diabetic medications were adjusted

**Fig. 3:**
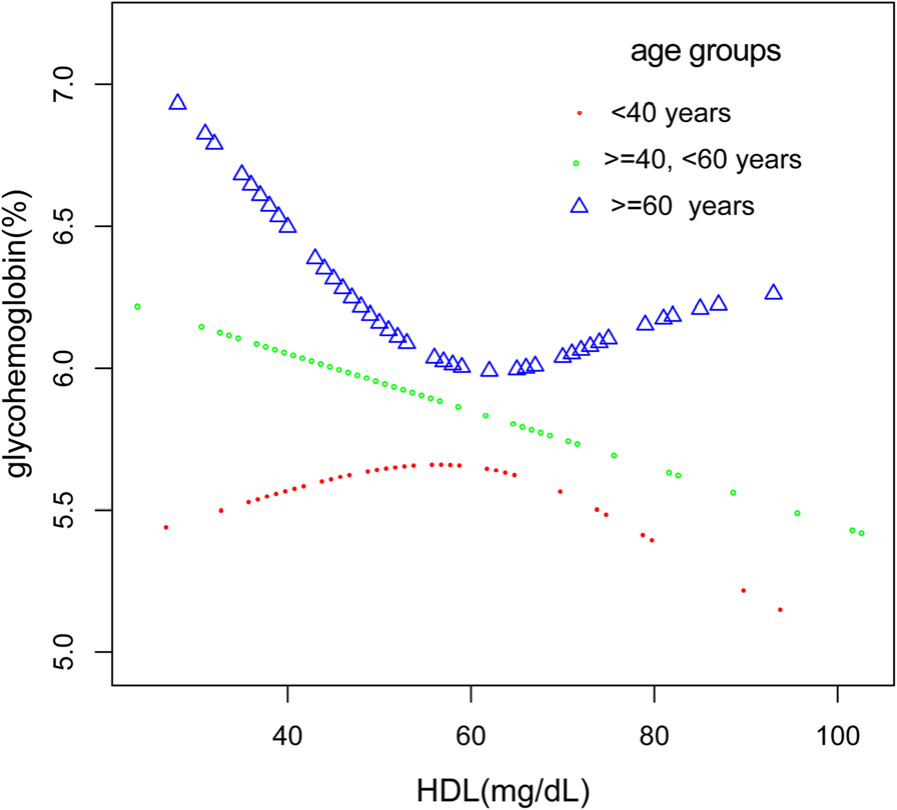
The association between HDL-C and glycohemoglobin stratified by age. Each line represents the smooth curve fit between variables. Sex, age, race/ethnicity, BMI, waist circumference, systolic blood pressure, diastolic blood pressure, ALT, Cr, TG, TC, LDL, FDG, γGT, uric acid, insulin, lipid-lowering medications and anti-diabetic medications were adjusted

Sex, age, race/ethnicity, BMI, waist circumference, systolic blood pressure, diastolic blood pressure, ALT, Cr, TG, TC, LDL, FDG, γGT, uric acid, insulin and lipid-lowering medications and anti-diabetic medications were adjusted in the interaction analysis:

Additionally, we also performed a weighed generalized additive model and a smooth curve fitting stratified by age and race/ethnicity to detect the non-linear association between HDL-C and glycohemoglobin in DM patients and further confirm the results. A U-shaped association was detected between HDL-C and glycosylated hemoglobin for people of other race/ethnicity or aged 60 and above eventually. Furthermore, we have observed an inverted U-shaped distribution between HDL-C and glycosylated hemoglobin in people under 40. Strangely and significantly, the inflection point calculated by a recursive algorithm of HDL-C in these groups was all 60 mg/dL. We further applied a two-piecewise linear regression model to examine HDL-C’s threshold effect on glycohemoglobin according to the smoothing plot (Table 3, Figs. 2-3).

**Table 3:** Threshold effect analysis of HDL-C and hemoglobin using two-precise linear regression

Glycohemoglobin	Adjustedβ(95% CI), *p*
**Age < 40 years**	
Fitting by a standard linear model	−0.000 (− 0.017, 0.016) 0.9675
Fitting by two precise linear model	
Inflection point	60
HDL-C < 60 mg/dL	0.034 (0.015, 0.053) 0.0030
HDL-C > 60 mg/dL	−0.082 (− 0.120, − 0.044) 0.0006
Log-likelihood ratio	< 0.001
**Age > =60 years**	
Fitting by a standard linear model	−0.016 (− 0.029, − 0.002) 0.0268
Fitting by two precise linear model	
Inflection point	60
HDL-C < 60 mg/dL	−0.043 (− 0.066, − 0.020) 0.0006
HDL-C > 60 mg/dL	0.012 (− 0.011, 0.035) 0.3178
Log-likelihood ratio	0.001
**Other race/ethnicity**	
Fitting by a standard linear model	0.007 (−0.012, 0.027) 0.4770
Fitting by two precise linear model	
Inflection point	60
HDL-C < 60 mg/dL	−0.002 (− 0.040, 0.036) 0.9181
HDL-C > 60 mg/dL	0.013 (−0.015, 0.041) 0.3823
Log-likelihood ratio	0.378

Sex, age, race/ethnicity, BMI, waist circumference, systolic blood pressure, diastolic blood pressure, ALT, Cr, TG, TC, LDL, FDG, γGT, uric acid, insulin and lipid-lowering medications and anti-diabetic medications were adjusted.

## Discussion

The most prominent finding to emerge from the analysis is that HDL-C was significantly negatively correlated with glycosylated hemoglobin, which, as far as we knew, is the first time to detect such a correlation in this population. Race/ethnicity and age were considered the most prominent interactive factors that affect the relationship between HDL and glycosylated hemoglobin by the interaction analysis. A U-shaped association was detected between HDL-C and glycosylated hemoglobin for people of other race/ethnicity or aged 60 and above, and the inflection point of HDL-C was 60 mg/dL. It is somewhat surprising that an inverted U-shaped distribution between HDL-C and glycosylated hemoglobin in people under 40 was detected, and the point of inflection of HDL-C was also located at 60 mg/dL.

HDL-C contains hundreds of lipids and proteins, which exerts much potential vascular protection and anti-diabetic effects on cells [24]. It was reported in the relevant literature that adults with low HDL-C, especially those with diabetes, have an increased risk of coronary heart disease and stroke [25]. HDL-C has been found to be inversely correlated with cardiovascular diseases, and its value as a predictor of cardiovascular risk remains largely unchallenged [16, 26–28]. A population-based cohort study conducted by Seung-Hwan Lee et al. [29] pointed out the low mean and high variability of HDL-C were independent predictors of diabetes and had an additive effect. Elevating and stabilizing HDL-C may be worthwhile to lessen the risk of diabetes.

As an indicator that can evaluate recent average glycemic control, glycated hemoglobin is also regarded as an essential sign of cardiovascular and cerebrovascular mortality and morbility in diabetic patients, which is of great significance [3, 4, 30]. Similar to the literature knowledge, Yun Shen et al .[31] found in their research that HDL-C was inversely associated with the risk of ischemic and hemorrhagic stroke among DM patients. It can be seen that it is important to evaluate the relationship between HDL-C and glycosylated hemoglobin in diabetic patients. At present, relevant research reports are rare. Recently, some scholars have assessed the relationship between HDL-C subtypes and glycosylated hemoglobin between 101 patients with metabolic syndrome and 77 healthy controls. The results showed that in those with metabolic syndrome, as the HbA1c level increased, pre-β1-HDL gradually increased, while HDL 2a gradually decreased [32]. However, in this cross sectional study, we found that HDL-C is significantly negatively correlated with glycosylated hemoglobin, which implies that lower HDL-C may be related to poorer glucose control in diabetic patients and may increase the risk of cardiovascular diseases.

Traditionally, the theory that increasing HDL-C to higher levels is more conducive to reducing cardiovascular diseases and adverse events is being questioned by researchers [33, 34]. Some scholars have even suggested that reducing HDL-C is more valuable in certain patients. Consistent with these theories, we found that for those who were 60 or older and those of other race/ethnicity, it may be best to maintain HDL-C within 60 mg/dl. In other words, though HDL-C reflects the synthesis of lipid metabolism, for some certain persons, such as diabetics, and it is not the higher, the better. However, this conclusion needs to be confirmed by a multi-center study with larger sample size. Another important finding of our research is that for young diabetic patients (younger than 40 years old), perhaps upholding HDL-C above 60 mg/dl is the best choice. This is similar to the findings of previous researchers. In a study that included 15,633 patients undergoing PCI, it was proved that there was a U-shaped association between HDL-C and overall mortality, with HDL-C levels of 30-50 mg/dl, which was the most favorable result. However, HDL-C levels < 30 mg/dl or > 50 mg/dl are associated with poor results [35]. Another study also found that HDL cholesterol level has nothing to do with coronary artery disease risk, while some qualitative characteristics of HDL (related to size, particle distribution, and cholesterol and triglyceride content) are related to CAD risk [36]. How to explain the contrasting results of age < 40 vs. age > =60 showing that the relationship between HDL-c and Hba1c is inversely and directly related. We thought that this may be related to different types of diabetes. Because the proportion of type 1 diabetes may be higher in people under 40 than people over 60. Unfortunately, because the NHANES questionnaire did not distinguish between different types of diabetes, we did not conduct subgroup analysis of different types of diabetes. This needs more research to confirm.

It is the first study to explore HDL-C and glycosylated hemoglobin in NHANES, providing significant reference value for managing dyslipidaemia, especially HDL-C, in diabetic patients and reducing cardiovascular and cerebrovascular diseases. Nevertheless, there are still some drawbacks of the present study: 1. Since this was a cross-sectional study, the subjects were not followed up, and the relationship between HDL-C and adverse outcomes and causality could not be effectively evaluated. 2. This study did not exclude patients with other diseases that may interfere with blood lipids. Scholars still need to be cautious when facing the results of the study. 3. Since the environment and living habits of people of different ages are nonidentical, this study spanned nearly 20 years and may cause more significant bias.4.We haven’t performed subgroup analysis stratified by Hba1c due to fewer cases of Hba1c > 7%, which might be another shortcoming of the present study. What’s more, because most individuals in the present study had glycosylated hemoglobin less than 6.5%, our conclusions seems to be more suitable for subjects with good glycemic control. The conclusions of this study still need to be confirmed by a larger sample size study.

## Conclusions

An apparent negative correlation between HDL-C and glycohemoglobin was discovered in the present research, which may indirectly imply that in addition to fasting glucose and glycosylated hemoglobin, HDL-C may be closely associated with glycemic control and is also crucial for diabetic patients. However, a U-shaped relationship was also observed in a certain kind of people, which implied that, though HDL-C is considered a metabolism and anti-atherogenic property, for diabetics, it is not the higher, the better.

## Supplementary Information

**Additional file 1.**

## Data Availability

The data used to support the findings of this study are available from corresponding author upon request.

## Abbreviations

HDL-C: High-density lipoprotein cholesterol
NHANES: National Health and Nutrition Examination Survey
NCHS: National Center for Health Statistics
SBP: Systolic blood pressure
DBP: Diastolic blood pressure
ALT: Alanine aminotransferase
Cr: Creatinine
TG: Triglyceride
TC: Total cholesterol
LDL_C: LDL-cholesterol
FDG: Plasma fasting glucose
γGT: Gamma-glutamyl transferase
sUA: Serum uric acid
MEC: Mobile Inspection Center
BMI: Body mass index
