# Supplementary material for: The relationship between high-density lipoprotein cholesterol (HDL-C) and glycosylated hemoglobin in diabetic patients aged 20 or above: a cross-sectional study
Source: BMC Endocr Disord. 2021 Oct 11;21:198. doi: 10.1186/s12902-021-00863-x (PMC8507179; doi:10.1186/s12902-021-00863-x)
Supplement: Supplementary file 1 — Additional file 1. [file 12902_2021_863_MOESM1_ESM.docx]

Laboratories and related equipments for tseting glycosylated hemoglobin and HDL -C

HDL-C was measured using a Hitachi 717 or Hitachi 912 (Roche Diagnostics, 9115 Hague Road, Indianapolis, IN 46250) in the 1999-2006 cycle in Johns Hopkins University and a Roche Modular P chemistry analyzer (Roche Diagnostics, 9115 Hague Road, Indianapolis, IN 46250) in the 2007-2012 cycle and the Roche modular P and Roche Cobas 6000 chemistry analyzers in the 2013-2018 cycle in the University of Minnesota. Glycohemoglobin was measured using Primus CLC330 and Primus CLC 385(Primus Corporation, Kansas City, MO) in the 1999 to 2004 cycle by the Diabetes Diagnostic Laboratory at the University of Missouri-Columbia and an A1c 2.2 Plus Glycohemoglobin Analyzer (Tosoh Medics, Inc., 347 Oyster Pt. Blvd., Suite 201, So. San Francisco, Ca 94080.) in the 2005 to 2006 cycle and an A1c G7 HPLC Glycohemoglobin Analyzer (Tosoh Medics, Inc., 347 Oyster Pt. Blvd., Suite 201, So. San Francisco, Ca 94080.) in the 2007-2018 cycle. In 2005-2012 the University of Minnesota, Minneapolis, MN performed this testing. However, in 2013-2018 the University of Missouri-Columbia began testing glycohemoglobin.
